# Water, sanitation and hygiene practices associated with improved height-for-age, weight-for-height and weight-for-age z-scores among under-five children in Nepal

**DOI:** 10.1186/s12887-020-2010-9

**Published:** 2020-03-23

**Authors:** Som Kumar Shrestha, Don Vicendese, Bircan Erbas

**Affiliations:** 1 London School of Hygiene & Tropical Medicine, London, England; 2 The Department of Mathematics and Statistics, La Trobe University, Melbourne, Australia

**Keywords:** Underweight, Stunting, Wasting, Children, WASH

## Abstract

**Background:**

Evidence of the influence of water, sanitation and hygiene (WASH) behaviors on childhood nutritional status is inconsistent. Few studies have examined their interactive effects. This study aimed to examine associations and interactions between WASH variables and preschool child undernutrition.

**Methods:**

Data from a nationally representative sample of 2352 children assessed during the 2016 Nepal Demographic and Health Survey were analyzed by multi-variable linear regression to understand the association between height-for-age (HAZ), weight-for-height (WHZ) and weight-for-age (WAZ) z-scores and WASH variables. Interactions between WASH variables, sex and area of residence on childhood nutritional status were also examined.

**Results:**

The mean z-score [standard deviation] for children’s WAZ, HAZ and WHZ scores were − 1.33 [1.1], − 1.52 [1.3] and − 0.65 [1.1], respectively. A unit increase in cluster sanitation coverage was associated with an increase of 0.30 (95%CI: 0.12 to 0.48) for WAZ and 0.28 (95%CI: 0.001 to 0.56) for HAZ scores. Household water purification practice was associated with an increase of 0.24 (95%CI: 0.07 to 0.41) in WHZ score. Handwashing practice with water and soap was associated with an increase of 0.15 (95%CI: 0.04 to 0.25) in WAZ and 0.13 (95%CI: 0.01 to 0.24) in WHZ scores. The effect of water purification practice was higher for rural areas compared to urban settings for HAZ scores (*p*-value for interaction = 0.02).

**Conclusions:**

Consistent with findings from other countries in the South Asian region, findings of this study highlight the potential importance of good WASH practices, and therefore the potential of WASH interventions, to contribute to improved nutritional status in rural Nepal.

## Background

Childhood under-nutrition is a major public health problem in developing countries. In 2018, globally, approximately 21.9% under-five children were estimated to be stunted and 7.3% children wasted, and almost two out of five stunted children belonged to South Asia [1]. Nepal is among countries having the highest prevalence of childhood undernutrition in the South Asian region [2]. A recent Nepal Demographic and Health Survey (NDHS) showed almost 36% of under five children were stunted, 10% were wasted and 27% were underweight in 2016 [3].

A public health programme for tackling the burden of under-nutrition in children has been a high priority for the Nepal Government. In 2011, Nepal joined the global movement Scaling Up Nutrition as its commitment to end malnutrition [4]. Realizing the importance of each sector’s potential to contribute to the national goal to reduce childhood undernutrition [5], the Multi-Sector Nutrition Plan (2013–2017) was endorsed and envisioned inter-sectoral collaboration between various sectors such as education, agriculture, health and water and sanitation. Several international donors have pledged their support to tackle childhood malnutrition through funding nutrition specific interventions delivered from health facilities and implementing nutrition sensitive interventions addressing underlying determinants of undernutrition [6–8]. Over the past decade, Nepal has made notable progress on improving underlying determinants of childhood nutrition such as education attainment, WASH coverage, maternal and child health and poverty reduction. The same period also coincides with a substantial reduction in childhood undernutrition, particularly for childhood stunting [3, 9]. Despite these successes, Nepal still lagged behind achieving the target of sustainable development goals for reducing the prevalence of stunting and underweight by 2017 [10]. Moreover, the recent national survey showed widespread disparities in the burden of childhood undernutrition across different socio-economic status, ethnic communities and geographical regions [3]. This shows that the current progress made in reducing childhood undernutrition has failed to benefit children from all population subgroups especially among the poor and vulnerable communities living in rural areas. Childhood undernutrition still remains one of the major public health challenges in Nepal.

Several underlying factors are associated with poor nutritional outcomes among children [11, 12] and the evidence on the adverse consequences of poor WASH practices on child’s nutrition wellbeing is also growing [13–15]. The most common hypothesis is that poor WASH facilities and practices mediate transmission of faecal pathogens that causes diarrhea [16–18], and diarrhea in turn exacerbates undernutrition [19, 20]. However, other authors have argued that the primary causal pathway for influence of WASH factors on childhood undernutrition is tropical enteropathy and therefore, the estimates modeled entirely through diarrhea could have underestimated the contribution of WASH factors on childhood undernutrition [21]. The relative contribution of WASH interventions on childhood undernutrition is still unresolved in the current literature [21, 22] with some studies providing conflicting results depending upon study design, nutritional outcomes and geographical settings where studies were conducted [11, 21–23]. Despite this, a majority of cross-sectional studies have consistently demonstrated a crucial role of improved WASH practices on childhood nutrition outcomes [6, 11, 24, 25]. However, wide spread variation in methodological approaches and study settings have often posed a considerable challenge for interpretation and synthesis of study findings, limiting the prospect of providing definitive policy recommendations. Studies illustrating how different WASH factors encompassing all important components, namely safe drinking water, improved sanitation and handwashing practices, are associated with different forms of undernutrition is limited in the current literature. The fact that different WASH components are known to be independently associated with child nutrition outcomes, indicates that failure to adjust for all WASH components could potentially bias study findings. On the other hand, WASH related variables could interact among themselves in a complex manner with differential impacts on nutrition outcomes [26]. Only a few studies have explored evidence of interaction among different WASH components on child’s nutrition outcomes [14, 23].

Therefore, this study aimed to determine the role of different components of WASH facilities on various forms of childhood undernutrition. Specifically, we intend to evaluate how WASH factors are associated with different nutritional outcomes, WAZ, HAZ and WHZ scores among under-five children. In addition, this study also explored whether combined WASH facilities delivered any synergistic benefit on different forms of nutrition outcomes and whether any synergistic benefit was evident across urban and rural settings and by child’s sex. Within these aims, we endeavored to find evidence to guide public health policy to scale-up WASH interventions to further accelerate the progress on tackling persistent undernutrition in developing countries.

## Methods

### Data source and sampling

This study analyzed cross-sectional data from the nationally representative sample of the NDHS (2016). A detailed description of the sampling methods and methodology is reported elsewhere [3]. Briefly, the sampling frame of the NDHS 2016 survey was the updated version of the National Population and Housing Census 2011 conducted in Nepal. Nepal consists of 77 districts distributed across three ecological zones (Mountain, Hill and Terai) and 7 provinces. Almost one-third (34%) of the population was under age 15 and under-five children account for almost 11% of total population. Multi-stage sampling was used and samples were selected in two stages for the rural areas and in three stages for the urban areas. Each province was segregated into urban and rural areas that yielded a total of 14 sampling strata. At the first stage, the wards were selected independently in each stratum using a probability proportional to size strategy. In the rural areas, the selected wards with a relatively small household number (average of 104 households) directly served as the primary sampling units (PSUs). In the urban area, wards were selected as the PSUs, however, due to their large size (average of 800 households), each ward was further stratified as Enumeration Areas (EA) and one EA was randomly selected from each PSU. In the second stage, a complete household listing was carried out from the selected sampling clusters (wards in rural and EAs in urban) that served as the sampling frame for the selection of households. Finally, the predetermined fixed number of 30 households was selected from each cluster with systematic random selection methods.

**Figure 1:**
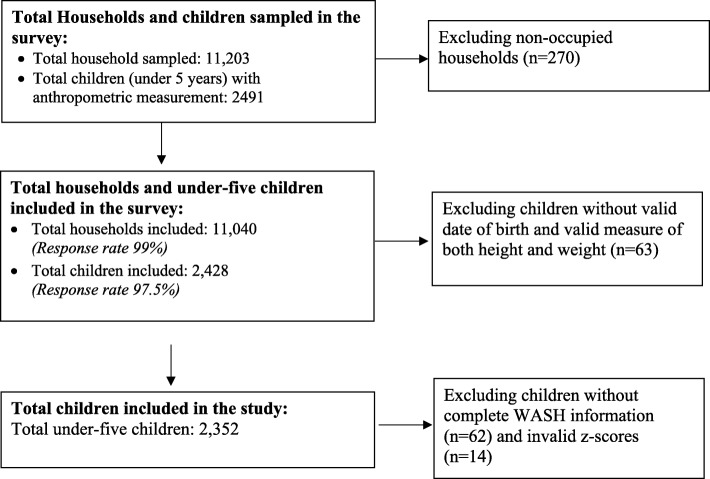
Fig. 1 Flow-chart for under-five children sampling procedure for NDHS, 2016

The total of 11,473 households from 383 sample wards or enumeration areas were selected for the survey. The final study comprised a total of 11,040 households with a response rate of 99% that included 2428 children below five years of age (Fig. 1: Flow-chart for under-five children sampling procedure for NDHS, 2016). The survey used a household questionnaire and observational checklist for collecting the information on household WASH facilities and practices.

### Study variables

#### Outcome variables

The variables for measuring child nutritional outcomes were based on anthropometric measures of children using an internationally recognized standard practice for assessment of malnutrition at population level [27]. The primary outcome variables were the z-scores for WAZ, HAZ and WHZ, calculated based on the median of the World Health Organization (WHO) reference population. The detail calculation for deriving z-scores is provided elsewhere [28]. Three nutrition outcomes measure different forms of childhood undernutrition. Stunting (low HAZ z-score) is regarded as an indication of long-term nutritional deficiency characterized by recurrent or chronic illness. Wasting (low WHZ z-score) is regarded as an indication of a recent event due to diarrhea or illness. Underweight (low WAZ z-score) represents both acute and chronic shortage of nutrition [29].

Children were dichotomously categorized with reference to the median of the WHO reference population [30]. Children with a z-score equal or above − 2 SD of the reference population were categorized as “adequately nourished” children and below − 2 SD as “undernourished” for all nutritional outcomes. We excluded anthropometric measurements with a z-score below − 6 SD and above + 6 SD as outliers.

#### Exposure variables

WASH related variables such as cluster sanitation coverage, water treatment practices and hand washing behaviors with soap and water, were the major exposure variables. Sanitation facilities could be of different types, constructed using different locally available materials. Their true benefit is only achieved depending on how effectively such facilities prevent direct exposure of immediate surroundings to human faeces. We categorized household sanitation facilities as either “improved”, which included facilities with sewer connections, septic system connections, pour-flush latrines, ventilated improved pit latrines and pit latrines with a slab or covered pit and “unimproved” for pit latrines without slabs or platforms or open pit, hanging latrines, bucket latrines and open defecation [31]. We defined community sanitation coverage as the percentage of households with improved sanitation facilities within sampled clusters.

Another important WASH variable is access to safe drinking water but the methods for how the safe drinking water variable is defined and included in existing nutritional studies vary markedly. They mainly differ on either use of a protected water source [24, 32], piped water source [11, 33] or household water treatment methods [34–36]. In resource constraint settings where a water pipeline system is unreliable and not well maintained, the risk of water contamination in the supply chain remains high [16, 37] potentially diluting the effect measure and strength of association. In Nepal too, most of the piped water system is not sufficiently well maintained and fully functional to deliver the expected services [38]. Therefore, we selected household water purification practice as a relatively reliable proxy indicator for safe drinking water without giving too much reliance on the safety of the water supply system.

Hand-washing practice with soap and water is an important WASH component but measuring actual hand washing practices at different critical points within the household level has always being challenging due to the following reasons. Firstly, hand washing practice is usually measured at different critical points that make it difficult for summarizing the behavior of individuals at household level. Secondly, the information on hand washing behavior is usually based on participants’ verbal responses and so may induce affirmative bias since participants are more likely to report positive behaviors than actual practice [39, 40]. Therefore to overcome these limitations, we included availability of soap and water measured through direct household observation as a proxy indicator for hand washing practices that addresses the need for monitoring hand washing behavior of all household participants and is also less subject to bias compared to verbal response.

#### Data analysis

A linear regression model was used to obtain β-coefficients that depict an increase in z-scores for WAZ, HAZ and WHZ associated with a unit increase in sanitation coverage. While for other WASH components (household water purification practices and availability of soap and water), they represented the difference in z-score associated with whether or not household practice included water treatment methods and hand-washing behaviors.

We conducted supplementary analyses using binary nutritional outcome variables to present the Odds Ratios (OR) for risk of childhood underweight, stunting and wasting associated with WASH variables to indicate if interpretation of our findings are robust and independent of the model selected for statistical analysis.

We adjusted for common confounding variables described in existing literature such as child’s birth weight, child’s age, child’s sex, wealth quintiles, use of clean fuel, sex of household head, ecological region, area of residence, women’s age, women's marital status, women’s education, women’s smoking status, women’s Body Mass Index (BMI), Antenatal Care Visit (ANC) 4th visits, institutional delivery, frequency of watching TV and child's diarrhea incidence over past 2 weeks (Supplementary Table 1).

The statistical significance level was set at a *p-*value less than 0.05. We used sampling weights to adjust for the complex survey design and non-proportionate selection probability. The interaction between different WASH variables, sex and area of residence on childhood undernutrition outcomes were also examined. For interaction analysis, we considered a *p*-value of < 0.1 as the level of significance to not miss important associations [41]. This study used Stata SE 14.1 (StrataCorp, College Station, Texas) for the analysis.

## Results

This study included a total of 2352 children after excluding samples with incomplete WASH information (*n* = 62) and either missing or outlier values for anthropometric measurements (*n* = 14).

### Household WASH and under-five children nutritional status

Household WASH status and the mean z-score for WAZ, HAZ and WHZ among under-five children are presented in Table 1. Among all households, 75.7% had improved sanitation, while average cluster sanitation coverage was 75.3%. Household water purification practice and availability of soap and water at the time of survey were 18.3% and 37.5% respectively. The mean z-scores for HAZ, WHZ and WAZ were − 1.52 [1.3], − 0.65 [1.1] and − 1.33 [1.1] respectively (Table 1). Among all children nationally, 35.8% were classified as stunted, 9.8% as wasted and 27% as underweight (Table 2).

**Table 1:** Household water sanitation and handwashing practices and mean z-score for underweight, stunting and wasting among under-five children (*n* = 2352)

Variables	Number (%)
**Proportion of household with improved toilet, water purification practices and availability of soap and water**	
Household with improved sanitation	1781(75.7%)
Cluster sanitation coverage percentage [SD]	75.3 [32]
Household with practice of water purification	430 (18.3%)
Household with soap and water available	882 (37.5%)
**Nutritional Outcome Indicators**	**Mean [SD]**
z-score for height-for-age	−1.52 [1.3]
z-score for weight-for-height	− 0.65 [1.1]
z-score for weight-for-age	−1.33 [1.1]

**Table 2:** Under-five children z-score classification for underweight, stunting and wasting (*n* = 2352)

Outcome indicators	**Undernourished Children N (%)**			**Adequately nourished Children N (%)**		
	z-score < − 3	z-score between − 3 to <− 2	Total (z- score < − 2)	z-score between − 2 to + 2	z-score > + 2	Total (z-score > − 2)
Height-for-age (Stunting)	284 (12.1)	559 (23.8)	843 (35.8)	1489 (63.3)	20 (0.85)	1509 (64.2)
Weight-for-height (Wasting)	44 (1.9)	187 (8)	231 (9.8)	2092 (88.9)	29 (1.2)	2121 (90.2)
Weight-for-age (Underweight)	124 (5.3)	511 (21.7)	635 (27.0)	1710 (72.7)	7 (0.3)	1717 (73.0)

### Association between WASH related variables and childhood under-nutrition status

Unadjusted and adjusted β-coefficients depicting the linear associations between WASH variables and childhood nutritional status are presented in Table 3. All WASH related factors were found to be significantly associated with indicators of nutritional status in the unadjusted models. In the adjusted model, the cluster sanitation coverage was associated with an increase of 0.28 (95%CI: 0.001 to 0.56) in HAZ score and 0.30 (95%CI: 0.12 to 0.48) in WAZ score. The association was not significant for WHZ (Table 3).

**Table 3:** Linear regression coefficient for association between WASH related variables and nutritional outcomes among under-five Nepalese children (*n* = 2320)

WASH Outcomes	Height-for-age (β^, 95%CI)		Weight-for-height (β^, 95%CI)		Weight-for-age (β^, 95%CI)	
	Unadjusted	Adjusted	Unadjusted	Adjusted	Unadjusted	Adjusted
**Sanitation Coverage**	0.54 (0.35, 0.74)***	0.28 (0.001, 0.56) ^a,*^	0.66 (0.50, 0.82)***	0.19 (− 0.04, 0.42) ^a^	0.78 (0.62, 0.94)***	0.30 (0.12, 0.48)^a,^ **
**Water Purification**						
Yes	0.37 (0.16, 0.59)**	−0.02 (− 0.24, 0.20) ^b^	0.52 (0.38, 0.67)***	0.24 (0.07, 0.41)^b,^ **	0.56 (0.38, 0.73)***	0.11 (−0.06, 0.28) ^b^
No (Ref)	1	1	1	1	1	1
**Water & Soap available**						
Yes	0.46 (0.32, 0.59)***	0.09 (−0.05, 0.22) ^c^	0.29 (0.18, 0.40)***	0.13 (0.01, 0.24)^c,^ *	0.47 (0.35, 0.58)***	0.15 (0.04, 0.25)^c,^ **
No (Ref)	1	1	1	1	1	1

^a^Adjusted for child’s birth weight, child’s age, child’s sex wealth quintiles, use of clean fuel, sex of household head, ecological region, area of residence, women’s age, women's marital status, women’s education, women’s smoking status, women’s BMI, ANC 4th visits, institutional delivery, frequency of watching TV, child's diarrhea incidence over past 2 weeks, household water purification practice and household water and soap availability

^b^Adjusted for child’s birth weight, child’s age, child’s sex wealth quintiles, use of clean fuel, sex of household head, ecological region, area of residence, women’s age, women's marital status, women’s education, women’s smoking status, women’s BMI, ANC 4th visits, institutional delivery, frequency of watching TV, child's diarrhea incidence over past 2 week, cluster sanitation coverage and household water and soap availability

^c^Adjusted for child’s birth weight, child’s age, child’s sex wealth quintiles, use of clean fuel, sex of household head, ecological region, area of residence, women’s age, women's marital status, women’s education, women’s smoking status, women’s BMI, ANC 4th visits, institutional delivery, frequency of watching TV, child's diarrhea incidence over past 2 weeks, cluster sanitation coverage and household water purification practice

*p < 0.05; **p < 0.01; ***p < 0.001

Households with water purification practice were associated with an increase of 0.24 (95%CI: 0.07 to 0.41) in WHZ score but no association was found for WAZ and HAZ scores (Table 3).

Handwashing practice was associated with an increase of 0.13 (95%CI: 0.01 to 0.24) in WHZ score and 0.15 (95%CI: 0.04 to 0.25) in WAZ score (Table 3).

### Interaction between WASH related variables and childhood undernutrition

We found no evidence of interaction between WASH variables on child’s WAZ, HAZ and WHZ scores (Table 4).

**Table 4:** Interaction between WASH related variables for childhood undernutrition (*n* = 2320)

	**Height-for-age**			**Weight-for-height**			**Weight-for-age**		
**Improved sanitation availability**^**a**^	**With water purification**	**Without water purification**	***p -*value for interaction**	**With water purification**	**Without water purification**	***p -*value for interaction**	**With water purification**	**Without water purification**	***p-*value for interaction**
	0.44 (−0.55, 1.43)	0.21 (−0.06, 0.48)	0.28	0.31 (−0.53, 1.15)	0.20 (− 0.04, 0.44)	0.55	0.67 (− 0.11, 1.45)	0.29 (0.11, 0.47)**	0.25
**Water purification**^**b**^	**With soap and water**	**Without soap and water**		**With soap and water**	**Without soap and water**		**With soap and water**	**Without soap and water**	
	0.07 (−0.21, 0.34)	− 0.10 (− 0.46, 0.26)	0.87	0.28 (0.05, 0.51)*	0.15 (− 0.09, 0.38)	0.28	0.22 (0.02, 0.44)*	− 0.05 (− 0.27, 0.18)	0.16
**Soap and water availability**^**c**^	**With improved sanitation**	**Without improved sanitation**		**With improved sanitation**	**Without improved sanitation**		**With improved sanitation**	**Without improved sanitation**	
	0.69 (−0.07, 0.21)	0.07 (−0.36, 0.49)	0.39	0.14 (0.01, 0.27)*	0.04 (−0.24, 0.31)	0.36	0.15 (0.04, 0.26)**	0.08 (−0.25, 0.40)	0.97

^a^Adjusted for child’s birth weight, child’s age, child’s sex wealth quintiles, use of clean fuel, sex of household head, ecological region, area of residence, women’s age, women's marital status, women’s education, women’s smoking status, women’s BMI, ANC 4th visits, institutional delivery, frequency of watching TV, child's diarrhea incidence over past 2 weeks and household water and soap availability

^b^ Adjusted for child’s birth weight, child’s age, child’s sex wealth quintiles, use of clean fuel, sex of household head, ecological region, area of residence, women’s age, women's marital status, women’s education, women’s smoking status, women’s BMI, ANC 4th visits, institutional delivery, frequency of watching TV, child's diarrhea incidence over past 2 weeks and household sanitation availability

^c^ Adjusted for child’s birth weight, child’s age, child’s sex wealth quintiles, use of clean fuel, sex of household head, ecological region, area of residence, women’s age, women's marital status, women’s education, women’s smoking status, women’s BMI, ANC 4th visits, institutional delivery, frequency of watching TV, child's diarrhea incidence over past 2 weeks and household water purification practice

*p < 0.05; **p < 0.01

There was no evidence of interaction between WASH variables and child’s sex for under-nutrition outcomes (result not shown). However, we observed a statistically significant interaction between improved water purification practice and area of residence on child’s HAZ score (*p*-value for interaction = 0.02) as shown in Table 5.

**Table 5:** Interaction between household water purification practices and area of residence on childhood stunting (*n* = 2320)

**Water purification practice**	**Height-for-age z-score**		
	**Urban**	**Rural**	***p*-value for interaction**
	−0.14 (− 0.41, 0.13) ^*a*^	0.19 (− 0.1, 0.5) ^*a*^	0.02*

^a^Adjusted for child’s birth weight, child’s age, child’s sex wealth quintiles, use of clean fuel, sex of household head, ecological region, area of residence, women’s age, women's marital status, women’s education, women’s smoking status, women’s BMI, ANC 4th visits, institutional delivery, frequency of watching TV, child's diarrhea incidence over past 2 week, cluster sanitation coverage and household water and soap availability

^*^p < 0.05

### Supplementary analysis

We did the supplementary analyses by modelling nutritional variables as dichotomous outcome variables. Household availability of soap and water was associated with decreased odds of childhood stunting (OR: 0.70, 95%CI 0.54 to 0.92). Sanitation coverage (OR: 0.53, 95%CI 0.28 to 0.99) and household water purification (OR: 0.39, 95%CI: 0.21 to 0.73) were associated with childhood wasting. The odds ratio of childhood underweight decreased significantly with a unit increase in sanitation coverage (OR: 0.59, 95%CI 0.38 to 0.93) and household availability of soap and water (OR: 0.65, 95%CI 0.51 to 0.84) (Supplementary Table 2).

## Discussion

This study found significant associations between WASH related factors and different forms of undernutrition among under-five children, both unadjusted and after controlling for potential confounders, however, the role of individual WASH factors differed substantially for different nutritional outcomes. Child HAZ score had a significant positive linear association with cluster sanitation coverage, while the WHZ score was positively linearly associated with household water purification practice and availability of soap and water. Increase in WAZ score was associated with increased sanitation coverage and household availability of soap and water. We found no evidence of interaction between different WASH variables on childhood nutrition outcomes. The influence of water purification practices on childhood stunting differed in urban and rural settings but child’s sex was not associated with WASH and undernutrition. 

Cluster sanitation coverage was associated with increased z-score for HAZ and WAZ and the findings are consistent with other studies that have evaluated the role of community sanitation on undernutrition [11, 13, 42]. Similar to our findings, previous studies from Nepal have also identified community sanitation coverage as a key determinant of improving HAZ score [11]. Other studies that examined the association between household sanitation and child nutrition status also reported similar conclusions [12, 23]. However, sanitation coverage is known to have positive externalities providing benefits beyond the household level and failure to adjust for community level sanitation could have underestimated overall benefits of sanitation on child’s nutritional outcomes [13, 42] in those studies.

The cluster sanitation coverage also had a significant influence on child’s WAZ score. A Mali study also showed a positive association where the benefit of sanitation coverage incremented child’s WAZ but leveled off after reaching 60% coverage. We found a positive linear association where the z-score for WAZ increased consistently with increase in sanitation coverage. Our study failed to find an association between sanitation coverage and WHZ z-score, in contrast to earlier studies in Nepal [11] and Bangladesh [43] where positive associations were reported. It should be noted that in the supplementary analysis where we modelled childhood wasting (dichotomized version of WHZ z-score using the WHO reference population), cluster sanitation coverage was significantly associated with decreased likelihood of wasting (Supplementary Table 2). These results suggest methodological differences in assumptions about how the outcome variable is defined and functional fit in statistical modeling could explain at least some discrepancies in study findings across different nutritional researches.

In our adjusted model, household water purification practice was significantly associated with increased z-score for WHZ but no evidence was observed for WAZ and HAZ. Prior nutritional studies from Nepal have mainly included piped water supply as an exposure variable [11, 24]. Our findings are consistent with those studies demonstrating an important association between safe drinking water and child’s WHZ z-score but not for HAZ [11, 24]. Households not having access to safe drinking water, either measured through household water purification practice or piped water supply, might have increased risk of water borne and infectious disease adversely affecting the child’s immediate nutritional wellbeing depicted in the form of wasting. However, water quality was not associated with z-score for HAZ and WAZ which indicates its effect being either neutralized for more severe forms of undernutrition or children experiencing catch-up growth with no major impact on long term nutritional health in a Nepalese context. Nevertheless, other studies have reported significant associations between water treatment practices and childhood stunting [35, 44]. The discrepancies in study findings could be due to various reasons such as cultural factors that determine water handling practices [45] and effectiveness of household water purification techniques [46] that determines the quality of water and thus leading to a varying effect on nutritional outcomes. The availability of sufficient water could be another important factor that affects household choices for adopting healthy WASH behaviors [15] as well as the risk of infectious diseases having adverse consequences on the nutritional status of children. These factors could partly explain discrepancies in study findings across different settings and locations.

We found a significant positive linear association between household hand washing practice with soap and water and child’s z-scores for WAZ and WHZ. We did not observe a significant association between hand washing behavior and child’s HAZ but the association was significant in the supplementary analysis that showed decreased odds of stunting associated with hand-washing behavior (Supplementary Table 2). In line with our findings, other studies have also reported significant associations between hand-washing behavior and childhood stunting [23, 47]. Despite this, intervention trials have failed to report significant effects of handwashing behavior on childhood stunting [36, 40]. Possible explanations include poor compliance and challenges associated with sustaining handwashing behavior [36] that could therefore omit nutritional benefits to children. Similarly, intervention studies are often resource intensive and so planned for a relatively shorter duration that may fail to capture longterm nutritional benefits [40].

No evidence of interaction was observed despite consistent associations between increased z-scores for all nutritional outcomes and the combined effect of WASH practices. In contrast, an Indian study showed a stronger inverse association between sanitation and stunting among households with handwashing practice compared to those without such practice [23]. The Indian study was based on a very large sample (*n* = 34,639) and failure to observe any evidence of interaction in our study could be due to insufficient power. However, we found a significant interaction between water purification practice and area of residence on HAZ score, indicating gain in HAZ score associated with water purification was higher in the rural areas compared to the urban settings (Table 5). In rural locations, households mainly depend on unreliable water sources with varying water flow/supply across different times of the year and increased risk of microbial contamination [48]. The water treatment practice in such settings could have offered better benefit for child growth. We observed no interaction between WASH related variables and sex of the child for different nutritional outcomes.

We primarily focused our study on child nutrition outcomes variables on a continuous scale (z-score of various nutrition outcomes). However, modelling dichotomous (binary) nutritional variables has also been done often in other nutritional studies [14, 42]. Therefore, we did the supplementary analysis modelling dichotomous nutritional outcome variables to assess if study findings are robust irrespective of choice of the statistical model. A majority of associations retained their statistical significance but some deviations were also observed. In the logistic regression model, the associations between hand-washing practice and stunting and sanitation coverage and wasting were found to be statistically significant. Whereas the association between sanitation coverage and stunting and hand-washing practice and childhood wasting lost their significance in the new model (Supplementary Table 2). As dichotomization may result in loss of information [49], so we emphasize that the model based on continuous outcome nutritional variables should be considered as a more effective approach for providing a true measure of association.

### Strengths and limitations

Using the nationally representative samples from Nepal, our study demonstrated important roles of various WASH components on different forms of childhood undernutrition. The study findings could be generalized to Nepal and possibly to other countries with similar settings. However, our study has some limitations; the cross-sectional nature of the data is limited to establish causality of the association. The final analysis included fewer samples due to missing values (*n* = 135) but we carried out multiple imputation to check if missingness of variables had any effect on the interpretation of our results but we found no substantial change (results not shown). Though, we controlled for most of the confounders as identified in other nutritional studies, the possibility of residual confounding due to other unknown factors could not be fully ignored.

Complementary feeding has an important role in child nutritional health [50, 51]. We did not adjust for complementary feeding practices in the final model since the information was limited only to 6–23 months old children. However, we conducted a sensitivity analysis which included the Minimum Acceptable Diet (MAD) by taking samples of 6–23 months old children (*n* = 744). This variable summarizes both minimum feeding frequency and diversity apart from breast-milk [52]. The analysis showed MAD itself was not significantly associated with nutritional outcomes, WAZ (β-coefficient: -0.02, 95%CI: − 0.15 to 0.19), HAZ (β-coefficient: -0.01, 95%CI: − 0.24 to 0.22) and WHZ (β-coefficient: -0.5, 95%CI: − 0.24 to 0.14). It did not alter interpretation of the significant associations between WASH variables and childhood nutrition outcomes (result not shown), except for hand washing behaviors which were not significantly associated with WHZ (β-coefficient = 0.12, 95%CI: − 0.07 to 0.31). Therefore, this indicates that not having adjusted for MAD could have overestimated the association between hand washing behaviors and child’s WHZ score.

## Conclusions

Our study showed that WASH variables had an important role in nutritional outcomes among under-five year old children and that the findings could have important policy implications. The association of particular WASH factors varied widely across different forms of nutrition outcomes. This calls for careful consideration of evidence at the local level before selecting specific WASH interventions or programs in an effort to improve childhood nutritional outcomes.

The combination of various WASH components led to higher gains in z-scores, but we could not be sure if strategies to combine different WASH components would deliver any synergistic effect on childhood nutrition. Further studies with robust study designs and sufficient power could elucidate further the potential impact of combining comprehensive WASH strategies on improving childhood nutrition status. They may also assist policy makers to make a comparative assessment of available evidence to address the pervasive burden of undernutrition in developing countries. However, public health policy should continue to target improving all domains of WASH components that are known to deliver wider societal benefits beyond health and nutrition outcomes.

## Supplementary information

**Additional file 1: Table S1.** Descriptive summary of variables adjusted in the final regression models (*n* = 2352).

**Additional file 2: Table S2.** Odds ratio of underweight, stunting and wasting associated with WASH variables (*n* = 2320).

## Data Availability

Dataset used in this study could be assessed from https://dhsprogram.com/data/dataset/Nepal_Standard-DHS_2016.cfm?flag=0

## Abbreviations

ANC: Antenatal Care Visit
BMI: Body Mass Index
CI: Confidence Interval
EA: Enumeration Areas
HAZ: Height-for-age Z-score
MAD: Minimum Acceptable Diet
NDHS: Nepal Demographic and Health Survey
OR: Odds Ratio
PSUs: Primary Sampling Units
WASH: Water, Sanitation and Hygiene
WAZ: Weight-for-age Z-score
WHO: World Health Organization
WHZ: Weight-for-height Z-score
