# Supplementary material for: Water, sanitation and hygiene practices associated with improved height-for-age, weight-for-height and weight-for-age z-scores among under-five children in Nepal
Source: BMC Pediatr. 2020 Mar 23;20:134. doi: 10.1186/s12887-020-2010-9 (PMC7092611; doi:10.1186/s12887-020-2010-9)
Supplement: Supplementary file 1 — Additional file 1: Table S1. Descriptive summary of variables adjusted in the final regression models (n = 2352). [file 12887_2020_2010_MOESM1_ESM.docx]

**Additional table 1: Descriptive summary of variables adjusted in the final regression models (n=2352)**

| **Explanatory variables** | **Number** | **(%)** |
| --- | --- | --- |
| **Ecological region** |  |  |
| Mountain | 165 | 7.0 |
| Hill | 861 | 36.6 |
| Terai | 1326 | 56.4 |
| **Types of place of residence** |  |  |
| Urban | 1240 | 52.7 |
| Rural | 1112 | 47.3 |
| **Household Wealth quintiles** |  |  |
| Poorest | 487 | 20.7 |
| Poorer | 513 | 21.8 |
| Middle | 532 | 22.6 |
| Richer | 510 | 21.7 |
| Richest | 310 | 13.2 |
| **Sex of household head** |  |  |
| Male | 1602 | 68.1 |
| Female | 750 | 31.9 |
| **Clean fuel** |  |  |
| Yes | 1804 | 76.7 |
| No | 548 | 23.3 |
| **Child’s age (months)** |  |  |
| 0-11 | 463 | 19.7 |
| 12-23 | 508 | 21.6 |
| 24- 35 | 447 | 19.0 |
| 36-47 | 473 | 20.1 |
| 48-59 | 461 | 19.6 |
| **Child’s sex** |  |  |
| Male | 1230 | 52.3 |
| Female | 1122 | 47.7 |
| **Number of living child** |  |  |
| 1 | 720 | 30.6 |
| 2 | 847 | 36.0 |
| 3 or more | 785 | 33.4 |
| **Birth weight of child** |  |  |
| >= 2500 gram | 1265 | 53.8 |
| <2500 gram | 179 | 7.6 |
| Not weighted at birth | 908 | 38.6 |
| **Ever breastfeed by women** |  |  |
| Yes | 2303 | 97.9 |
| No | 49 | 2.1 |
| **Women age group** |  |  |
| 15-19 | 195 | 8.3 |
| 20-24 | 804 | 34.2 |
| 25-29 | 767 | 32.6 |
| 30-34 | 369 | 15.7 |
| 35-39 | 146 | 6.2 |
| 40 or more | 71 | 3.0 |
| **Marital status of women** |  |  |
| Currently married | 2338 | 99.4 |
| Formerly married | 14 | 0.6 |
| **Women’s education level** |  |  |
| No Education | 816 | 34.7 |
| Primary Education | 470 | 20.0 |
| Some Secondary | 565 | 24.0 |
| SLC and Above | 501 | 21.3 |
| **Women’s smoking Status** |  |  |
| No | 2300 | 97.8 |
| Yes | 52 | 2.2 |
| **Women’s Nutrition Status** |  |  |
| Normal | 1900 | 80.8 |
| Undernutrition | 452 | 19.2 |
| **Completed 4^th^ ANC visits** |  |  |
| No | 729 | 31.0 |
| Yes | 1623 | 69.0 |
| **Institutional delivery** |  |  |
| No | 1077 | 45.8 |
| Yes | 1275 | 54.2 |
| **Watching TV over last week** |  |  |
| Not at all | 901 | 38.3 |
| Less than once a week | 492 | 20.9 |
| Once a week or more | 960 | 40.8 |
