# Supplementary material for: Water, sanitation and hygiene practices associated with improved height-for-age, weight-for-height and weight-for-age z-scores among under-five children in Nepal
Source: BMC Pediatr. 2020 Mar 23;20:134. doi: 10.1186/s12887-020-2010-9 (PMC7092611; doi:10.1186/s12887-020-2010-9)
Supplement: Supplementary file 2 — Additional file 2: Table S2. Odds ratio of underweight, stunting and wasting associated with WASH variables (n = 2320). [file 12887_2020_2010_MOESM2_ESM.docx]

**Supplementary table 2: Odds ratio of underweight, stunting and wasting associated with WASH variables (n=2320)**

| **Sanitation Variables** | **Stunting (OR, 95%CI)** | | **Wasting (OR, 95%CI)** | | **Underweight (OR, 95%CI)** | |
| --- | --- | --- | --- | --- | --- | --- |
|  | **Unadjusted** | **Adjusted** | **Unadjusted** | **Adjusted** | **Unadjusted** | **Adjusted** |
| **Sanitation coverage** | 0.47 (0.34, 0.64)*** | 0.74 (0.47, 1.16)^a^ | 0.38 (0.26, 0.57)*** | 0.53 (0.28, 0.99) ^a, *^ | 0.31 (0.21, 0.44)*** | 0.59 (0.38, 0.93)^a,^* |
| **Water Purification** |  |  |  |  |  |  |
| Yes | 0.55 (0.39, 0.78)*** | 0.98 (0.65, 1.48) ^b^ | 0.29 (0.16, 0.50)*** | 0.39 (0.21, 0.73) ^b,^** | 0.42 (0.28, 0.63)*** | 1.07 (0.65, 1.75) ^b^ |
| No (Reference) | 1 | 1 | 1 | 1 | 1 | 1 |
| **Water & Soap available** ^c^ |  |  |  |  |  |  |
| Yes | 0.47 (0.38, 0.58)*** | 0.70 (0.54, 0.92)^c^,** | 0.66 (0.48, 0.91)*** | 0.82 (0.56, 1.20)^c^ | 0.40 (0.32, 0.52)*** | 0.65 (0.51, 0.84)^c^,** |
| No (Reference) | 1 | 1 | 1 | 1 | 1 | 1 |

*^a^Adjusted for child’s birth weight, child’s age, child’s sex wealth quintiles, use of clean fuel, sex of household head, ecological region, area of residence, women’s age, women marital status, women’s education, women’s smoking status, women’s BMI, ANC 4th visits, institutional delivery, frequency of watching TV, childhood diarrhea over past 2 weeks, household water purification practice and household water and soap availability.*

*^b^Adjusted for child’s birth weight, child’s age, child’s sex wealth quintiles, use of clean fuel, sex of household head, ecological region, area of residence, women’s age, women marital status, women’s education, women’s smoking status, women’s BMI, ANC 4th visits, institutional delivery, frequency of watching TV, childhood diarrhea over past 2 week, cluster sanitation coverage and household water and soap availability.*

*^c^Adjusted for child’s birth weight, child’s age, child’s sex wealth quintiles, use of clean fuel, sex of household head, ecological region, area of residence, women’s age, women marital status, women’s education, women’s smoking status, women’s BMI, ANC 4th visits, institutional delivery, frequency of watching TV, childhood diarrhea over past 2 weeks, cluster sanitation coverage and household water purification practice.*

**p<0.05; **p<0.01; ***p<0.001*
